# Modulation of pulmonary immune functions by the *Pseudomonas aeruginosa* secondary metabolite pyocyanin

**DOI:** 10.3389/fimmu.2025.1550724

**Published:** 2025-03-24

**Authors:** Shi Qian Lew, Sook Yin Chong, Gee W. Lau

**Affiliations:** Department of Pathobiology, University of Illinois at Urbana-Champaign, Urbana, IL, United States

**Keywords:** *Pseudomonas aeruginosa*, pyocyanin, reactive oxygen and nitrogen species, oxidative stress, immune modulation, chronic lung diseases

## Abstract

*Pseudomonas aeruginosa* is a prevalent opportunistic Gram-negative bacterial pathogen. One of its key virulence factors is pyocyanin, a redox-active phenazine secondary metabolite that plays a crucial role in the establishment and persistence of chronic infections. This review provides a synopsis of the mechanisms through which pyocyanin exacerbates pulmonary infections. Pyocyanin induces oxidative stress by generating reactive oxygen and nitrogen species which disrupt essential defense mechanisms in respiratory epithelium. Pyocyanin increases airway barrier permeability and facilitates bacterial invasion. Pyocyanin also impairs mucociliary clearance by damaging ciliary function, resulting in mucus accumulation and airway obstruction. Furthermore, it modulates immune responses by promoting the production of pro-inflammatory cytokines, accelerating neutrophil apoptosis, and inducing excessive neutrophil extracellular trap formation, which exacerbates lung tissue damage. Additionally, pyocyanin disrupts macrophage phagocytic function, hindering the clearance of apoptotic cells and perpetuating inflammation. It also triggers mucus hypersecretion by inactivating the transcription factor FOXA2 and enhancing the IL-4/IL-13-STAT6 and EGFR-AKT/ERK1/2 signaling pathways, leading to goblet cell metaplasia and increased mucin production. Insights into the role of pyocyanin in *P. aeruginosa* infections may reveal potential therapeutic strategies to alleviate the severity of infections in chronic respiratory diseases including cystic fibrosis (CF) and chronic obstructive pulmonary disease (COPD).

## 1 Introduction

*Pseudomonas aeruginosa* is a ubiquitous Gram-negative bacterium in the environment and a major pathogen in individuals with chronic pulmonary diseases and immunocompromised health conditions. Pulmonary infections are a significant global health concern, especially in immunocompromised individuals and patients with preexisting respiratory diseases such as cystic fibrosis (CF), chronic obstructive pulmonary disease (COPD), primary ciliary dyskinesia, and chronic bronchitis (1), leading to high morbidity and mortality rates. *P. aeruginosa* is especially prevalent in CF patients, with infection rates rising from 20% in infants to nearly 80-90% in adulthood (2). Prior to the highly effective modulator therapy (HEMT), CF patients faced a worse prognosis due to chronic colonization and infection, particularly with *P. aeruginosa*, which is associated with increased mortality and poorer long-term survival outcomes (3, 4). HEMT (e.g., Trikafta) has significantly improved lung function in CF patients by restoring airway mucus homeostasis (5, 6). However, its long-term impact on the population dynamics of *P. aeruginosa* and other CF pathogens remains unclear. The economic impact of *P. aeruginosa* infections is substantial, with patients who developed nosocomial infections experienced a 66% increase in healthcare costs compared to those infected by other bacterial pathogens (7), which greatly impact the healthcare system. The significant economic burden of *P. aeruginosa* infections is further exacerbated by the emergence of multidrug-resistant (MDR) strains, with hospitalization costs for MDR cases being three times higher than those with non-resistant infections (8). *P. aeruginosa* infections often lead to further complications, including pneumonia and infections at multiple body sites. Some key factors that drive *P. aeruginosa* as a major pathogen include its ability to express a plethora of quorum sensing (QS)-regulated virulence factors, multiple drug efflux pumps, as well as its ability to form biofilms, which significantly enhance its resistance to antimicrobials, making infections more challenging to eradicate (9–11). QS allows bacteria to sense population density through the release of autoinducers, triggering the expression of virulence genes. In *P. aeruginosa*, the QS system consists of the LasI-LasR and RhlI-RhlR circuits, which utilize acylated homoserine lactones (Acyl-HSL) such as C4-HSL and 3Oc12-HSL to regulate gene expression. The PqsR (also known as MvfR), a LysR-type transcriptional regulator, is responsive to the Pseudomonas quinolone system (PQS) that integrates into this network by modulating quinolone synthesis and interacts with the Las and Rhl systems to regulate the secretion of virulence factors (12–17). QS-regulated *P. aeruginosa* virulence factors include, among others, phenazines (18), Exotoxin A (19), proteases (20), and phospholipases (21).

**Figure 1:**
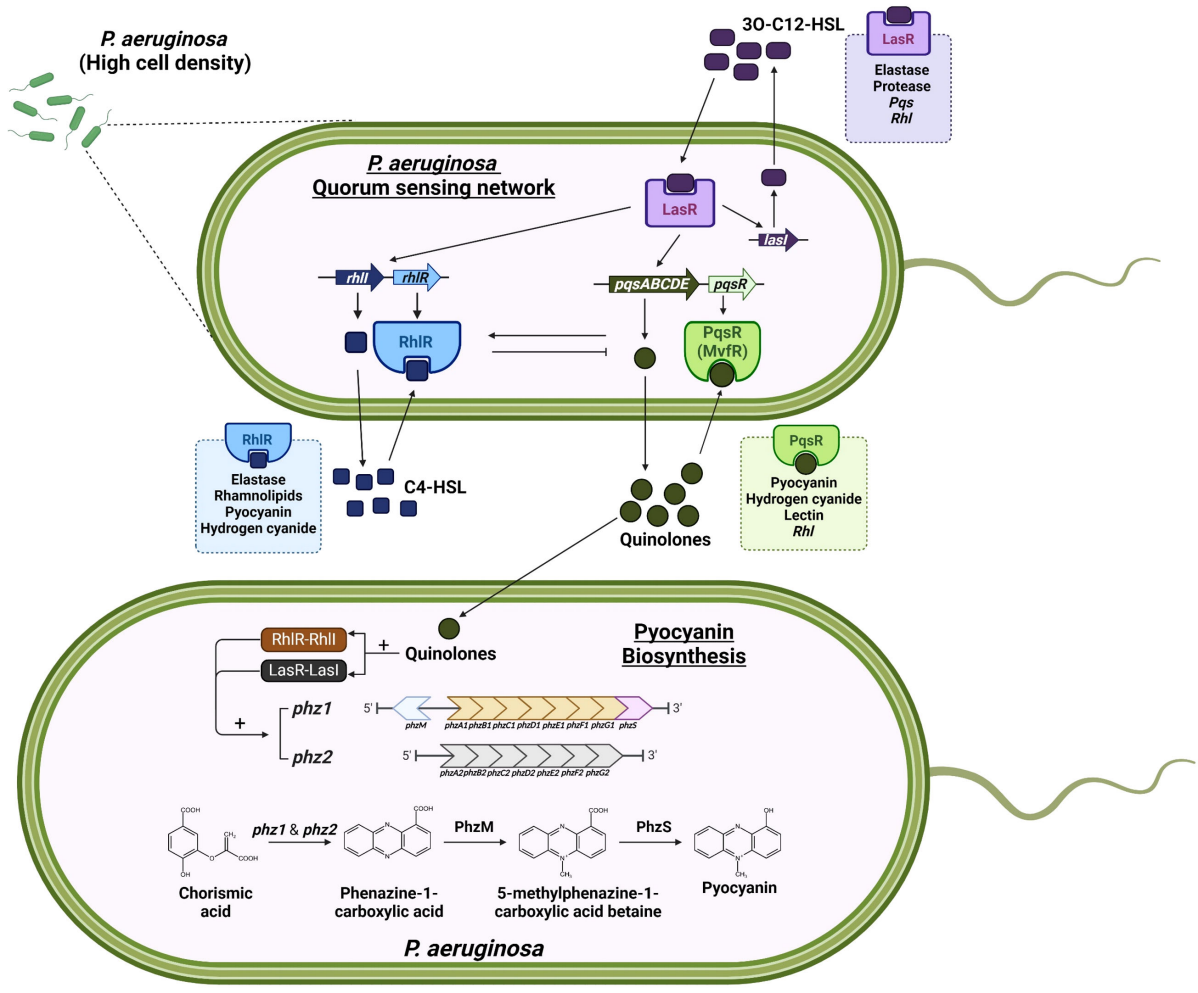
P*. aeruginosa* quorum sensing network and pyocyanin biosynthesis. Pyocyanin biosynthesis is regulated by the quorum sensing (QS) molecule quinolones and the QS regulatory system LasR-LasI, RhlR-RhlI and PqsR (MvfR). At the threshold population bacterial density, *phz1* and *phz2* operons are activated. This leads to the production of phenazine biosynthetic enzymes that convert chorismic acid into pyocyanin. Figure generated with BioRender.com.

One of the most well-studied virulence factors is the phenazines pyocyanin, a bluish pigment secondary metabolite that enhances the ability of *P. aeruginosa* to promote chronic lung infection (22). The biosynthesis of pyocyanin begins with the conversion of precursor chorismic acid, an intermediate metabolite that is utilized by microorganisms, through both *phzA1B1C1D1E1F1G1* (*phz1*) and *phzA2B2C2D2E2F2G2* (*phz2*) operons, and the *phzM* and *phzS* genes. These operons are regulated by the *P. aeruginosa* QS molecule quinolone. At high cell density, quinolones are secreted and detected by PqsR, which triggers the transcription of the *phz* operons, where the chorismic acid is modified into a tricyclic compound, and eventually, pyocyanin (**Figure 1**) (22–24). Pyocyanin is a zwitterion that is capable of diffusing through cell membranes freely. Previous studies have found pyocyanin in millimolar concentrations in the sputum of individuals infected with *P. aeruginosa* (25), with 16.5 μg/ml and 27 μg/ml recovered from bronchiectasis patients and CF patients, respectively (26, 27). Although 27 µg/ml is the highest amount of pyocyanin retrieved from human sputum, some studies have employed pyocyanin concentrations exceeding 100 µg/ml on human lung bronchial epithelial cells, raising concerns about the clinical relevance (28).

Pyocyanin is a redox-active tricyclic molecule structurally characterized by its nitrogen-containing heterocyclic core (29, 30). The redox cycling capabilities in pyocyanin enable it to become a potent inducer of oxidative stress that can reduce molecular oxygen quickly and generate reactive oxygen species (ROS) (31), primarily superoxide (O_2_•^-^) and hydrogen peroxide (H_2_O_2_); and reactive nitrogen species peroxynitrite (ONOO^-^). This effect significantly contributes to pathogenic outcomes, such as DNA damage (32), and disruption of membrane potential and redox balance in the host (33). In addition to damaging cellular structures, pyocyanin also activates several signaling pathways that promote inflammation, creating a vicious cycle of oxidative stress and immune dysregulation. Interestingly, *P. aeruginosa* has evolved multiple mechanisms to withstand the ROS effects generated by pyocyanin.​ The bacterium expresses various anti-oxidative enzymes, including superoxide dismutases (SOD), catalases, and peroxidases, that play a vital role in detoxifying the ROS produced by pyocyanin (34–37). These enzymes detoxify ROS into less harmful substances, such as water and oxygen. For instance, SOD catalyze the conversion of superoxide radicals into hydrogen peroxide, which is subsequently broken down into water and oxygen by catalases.

Research on pyocyanin has explored its virulence mechanism and interactions across various *in vivo* and *in vitro* models. *In vivo* model included diverse organisms such as the nematode *Caenorhabditis elegans* (18), the *Arabidopsis thaliana* leaf infiltration model (38), *Galleria mellonella* larvae (39) and brine shrimp *Artemia salina* (40). Additional more complex mammalian models include mouse strains such as C57BL/6J, BALB/c, and FVBN (41–43), and studies examining immunomodulatory effect of pyocyanin in sheep airways (44). *In vitro* studies using human cell lines have been extensively used to study pyocyanin, including NCI-H292 pulmonary carcinoma cells, 16HBE and NHBE (human bronchial epithelial cells), and A549 lung carcinoma epithelial cells (45, 46). Furthermore, pyocyanin exhibited cytotoxic effects against cancer cell lines such as SK-MEL-30 human melanoma and HT-29 human colon cancer cells, suggesting its potential as a cancer treatment (45). Unlike traditional 2D models, which lack the complexity of tissue architecture, 3D models such as the air-liquid interface (ALI) model of airway epithelial cells or lung organoids may provide a more realistic environment for assessing molecular modulation of pulmonary functions by pyocyanin and potential therapeutic effect in lung diseases by better replicating human lung tissue architecture and cellular interactions (47, 48). While *in vitro* studies provide controlled insights into cellular mechanisms and therapeutic effects, *in vivo* animal models with an intact immune system offer a comprehensive understanding of the biological impact of pyocyanin. Given the critical role of pyocyanin in *P. aeruginosa* pathogenesis, it is essential to understand the mechanisms by which this virulence factor disrupts pulmonary function and modulates the host immune response. In this article, we provide a critical synopsis on modulation of pulmonary immune response by pyocyanin.

## 2 Pyocyanin and oxidative stress in pulmonary cells

**Figure 2:**
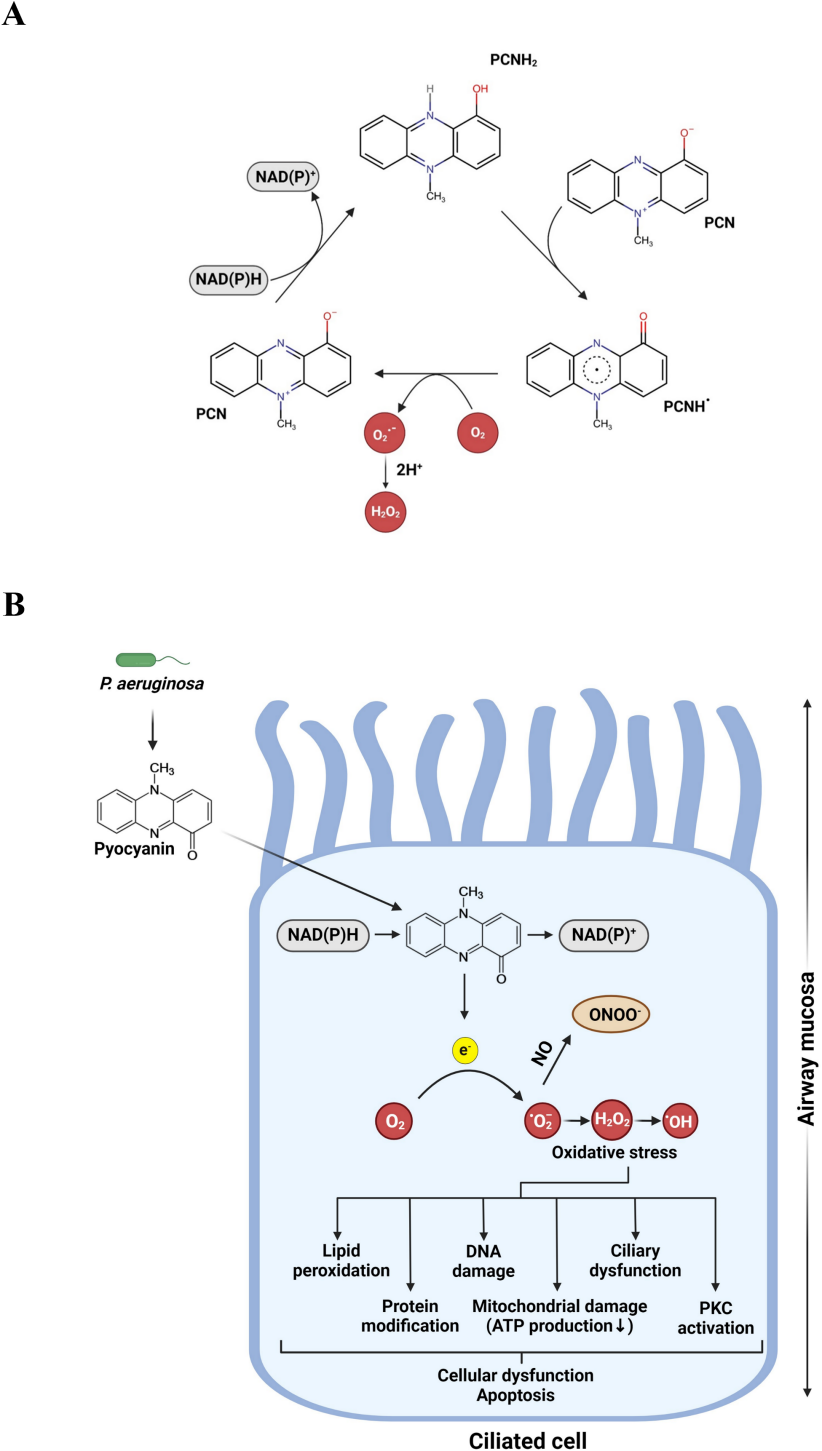
**(A)** Proposed mechanism of the pyocyanin (PCN) redox cycling. PCN can be reduced by NAD(P)H to form PCNH_2_. This reduced form can then be oxidized back to PCN by oxygen, generating an oxygen radical as a byproduct which is subsequently converted into hydrogen peroxide (H_2_O_2_). **(B)** Molecular basis of pyocyanin toxicity. Pyocyanin undergoes redox cycling by oxidizing NADPH to NADP^+^, generating reactive oxygen species (ROS) and reactive nitrogen species (RNS), contributing to the pathogenicity of the bacteria. Figures generated with BioRender.com.

Pyocyanin has a planar aromatic structure with conjugated double bonds that allow the displacement of electrons and facilitates in redox reactions. The phenazine rings can exist in different oxidation states, allowing pyocyanin to accept or donate electrons and cycling between reduced and oxidized states (29). This redox cycling process generates ROS, the primary mechanism underlying the toxicity of pyocyanin. Pyocyanin acts as a potent catalyst in cellular redox reactions, accepting electrons from NADH and NADPH and subsequently transferring them to molecular oxygen (O_2_). This process results in the formation of superoxide anions (O_2_^-^). These superoxide anions are then converted into hydrogen peroxide (H_2_O_2_) by superoxide dismutase (25, 49–52). Hydrogen peroxide can further undergo Fenton reactions, leading to the production of hydroxyl radicals (OH•), which are among the most damaging forms of ROS (53). In addition, O_2_^-^ can interact with NO to produce reactive nitrogen species (RNS), such as the highly toxic peroxynitrite (**Figure 2A**) (54). Collectively, these ROS and RNS generated by pyocyanin contribute significantly to several detrimental cellular processes. These harmful effects include lipid peroxidation, protein modification, mitochondrial and DNA damage, and ciliary dysfunction. Ultimately, the accumulation of these damaging processes results in overall cellular dysfunction and apoptosis (46, 55–58) (**Figure 2B**). The disruption of redox balance also inhibits crucial functions such as the dual oxidase-based antimicrobial system (DUOX) and ATP synthesis (45, 59). This interference facilitates bacterial persistence and contributes to chronic lung inflammation in individuals with CF and COPD (60).

### 2.1 Disruption of airway epithelial cells functions

The pulmonary epithelium serves as the first line of defense against inhaled pathogens and harmful particles, acting as a physical barrier, and an integral part of the innate immune system (61–63). However, the accumulation of ROS induced by the pyocyanin can significantly damage alveolar epithelial cells and subsequently disrupt the respiratory epithelial barrier function. This compromises the integrity of the lungs, making them more susceptible to infection and inflammation (64). In this section, we will explore the effects that pyocyanin-derived ROS exerted on the components of the airway immune system.

#### 2.1.1 Disruption of the airway mucociliary function

One critical consequence of pyocyanin-induced ROS production is the disruption of ciliary function in respiratory epithelial cells (65). The mucociliary clearance serves as the primary innate defense in clearing inhaled particles, pollutants, pathogens, and debris from the respiratory tract through coordinated beating (66, 67). Pyocyanin disrupts ciliary function by decreasing intracellular ATP levels, a crucial energy source for ciliary beating. This reduction in ATP level occurs as pyocyanin undergoes intracellular redox cycling, generating superoxide by depleting NADH. The release of ROS, which damages mitochondrial function and leads to ATP depletion, impairs the energy supply needed for proper ciliary movement (68–70). Dyneins are a family of cytoskeletal motor proteins that drive ciliary beating by converting the chemical energy stored in ATP into mechanical work with the help of dynein ATPases. Inadequate ATP supplies compromise the effectiveness of dynein-dependent ciliary motility (71, 72). This mechanism directly links the oxidative stress effects of pyocyanin to the impairment of ciliary function, contributing to defective mucociliary clearance and the persistence of respiratory infections in diseased lungs, such as CF and advanced stages of COPD (73). Furthermore, hydrogen peroxide has been shown to activate protein kinase C (PKC), which in turn leads to a reduction in the airway ciliary beat frequency. Research has also highlighted the phosphorylation of a key membrane-associated polypeptide, p37, by PKC, with phosphorylation occurring on both serine and tyrosine residues, decreasing ciliary beat frequency (74, 75). Additional studies have identified the biochemical pathways through which PKC regulates ciliary function, particularly in response to oxidative stress, aging, and alcohol consumption, all of which contribute to slowing ciliary beat frequency (76–78). These findings emphasize the significant role of PKC activation in mediating reduced ciliary activity in response to oxidative stress. The impairment of mucociliary function caused by pyocyanin-induced ROS leads to the accumulation of mucus, creating a niche for chronic microbial infection and vicious cycles of inflammation (79), particularly relevant in chronic respiratory diseases such as CF and COPD, where *P. aeruginosa* pyocyanin is expressed in abundance (79). The importance of cellular redox balance on ciliary function is further illustrated in the case of respiratory syncytial virus (RSV) infections, which the imbalanced ROS production in the airway epithelial microenvironment not only leads to oxidative damage to cilia but also disrupt the function of several redox-sensitive proteins such as protein kinase A (PKA), PKC, and protein phosphatase 1, which play crucial roles in regulating ciliary motility, eventually exacerbating airway mucociliary dysfunction (65, 80–83).

#### 2.1.2 Disruption of pulmonary epithelial barrier functions

The epithelial layer forms a crucial physical barrier in the intestinal, respiratory, and skin tissues, which play a pivotal role in maintaining tissue homeostasis, immune response, and protecting the body from pathogens. Disruption of the barrier function by oxidative stress and ROS, including those generated by pyocyanin, impairs mucosal immunity (84, 85). This is partly due to the impact on the actin cytoskeletal network in epithelial cells, which is crucial for maintaining cell shape, adhesion, and motility. However, ROS alters actin polymerization and stability, which disrupts epithelial cell barrier functions. One example is the ROS-induced modifications at Cys-374 of β-actin create disulfide bonds between actin monomers and prevent actin filament formation (86, 87). As a result, the ability of actin filaments to form a cytoskeleton is compromised, which causes cells to be unable to maintain their shape and integrity, contributing to “leaky” epithelial barriers that allow pathogens to invade host tissues. Furthermore, epithelial cells are held together by tight junctions (TJs) and adherens junctions (AJs), both essential for maintaining epithelial barrier integrity. ROS can disrupt these junctions, resulting in a compromised mucosal immune barrier. TJs are composed of claudins, occludins, and zonula occludens-1 (ZO-1) proteins, preventing paracellular leakage between epithelial cells (88). Under oxidative stress, ROS modifies occludins and claudins and causes the formation of disulfide bonds, impairing their ability in forming tight junctions and weakening the barrier (89). Besides TJs, ROS can also alter the integrity of AJs that mediate cell-cell adhesion and contact. AJs are characterized by cadherin cell adhesion transmembrane receptors that bind to each other in the intercellular space, establishing a physical connection between cells. Under physiological conditions, cadherins associate with catenins, which bind to cytoskeletal components such as actin filaments and microtubules, providing mechanical support to the cell (90, 91). In the case of AJs, ROS activate Src kinase, which phosphorylates p120-catenin. Phosphorylated p120-catenin dissociates from N-cadherin, causing internalization of N-cadherin from the membrane (87, 92). This leads to the disruption of adherens junctions, further impairing cell-cell adhesion and compromising the epithelial barrier, rendering host tissue susceptible to pathogen invasion.

Under oxidative stress, ROS can modify immune-related proteins, affecting their signaling and response to infections. For example, exposure to pyocyanin mediated ROS to human airway epithelial cell activate NF-κB and other inflammatory signaling pathways, leading to the production of pro-inflammatory cytokines including tumor necrosis factor-alpha (TNF-α) and interleukins (IL-1β, IL-6, IL-8) (93). These cytokines promote inflammation and disrupt the mucosal barrier, leading to increased epithelial permeability and immune cell infiltration. Research using cell culture models such as Calu-3 airway epithelial cells, has demonstrated that these cytokines affect the integrity and permeability of pulmonary epithelial barrier integrity by regulating key TJs proteins like occludin, claudin-2, and ZO-1. These processes are driven by the activation of the epidermal growth factor receptor (EGFR) and the ERK1/2 MAPK signaling pathway. This signaling not only increases the permeability between cells but also contributes to the breakdown of tight junctions, weakening the barrier in the airways (94–97). Inhibitors targeting EGFR or the ERK1/2 MAPK pathway were shown to attenuate cytokine-induced degradation of tight junction proteins (31). The disruption of tight junctions by pyocyanin not only weakens the epithelial barrier but also promotes *P. aeruginosa* invasion, where it exacerbates chronic lung infection in CF and COPD.

### 2.2 Disruption of systemic immunity functions

#### 2.2.1 Induction of neutrophil apoptosis

Neutrophils are among the first immune cell population responding to microbial invasion. Upon detection, neutrophils move rapidly to the infection site to phagocytize and destroy these pathogens. Pyocyanin is strongly neutrophilic. Mechanistically, ROS generated by pyocyanin triggers the release of pro-inflammatory cytokines such as the neutrophil chemotactic factor IL-8 in a NF-κB-dependent manner through the MAPK/ERK signaling (98, 99), further contributing to the pathological effects observed during *P. aeruginosa* infections (99–102), which will be further discussed in Section 2.3. This rapid and excessive influx of neutrophils leads to persistent inflammation and damage to the infected tissue by releasing ROS, proteinase (such as elastase and cathepsin G), myeloperoxidase, and metalloproteinases (103, 104). Anti-inflammatory drugs like glucocorticoids have been shown to limit the pro-inflammatory effects of neutrophils by downregulating key inflammatory factors, including IL-1β, TNF-α, and IL-8, thus diminishing the neutrophil mediated proinflammatory damage caused by neutrophilic stimuli such as pyocyanin (105, 106).

In addition to being neutrophilic, pyocyanin has been shown to accelerate neutrophil apoptosis (107–110). Studies have shown that pyocyanin generates excessive mitochondrial ROS in neutrophils (107), damaging mitochondrial function and contributing to neutrophil apoptosis. The premature death of neutrophils (10-fold increase in apoptosis within five hours post-exposure to pyocyanin) attenuates their antimicrobial activity and contributes to the persistence of *P. aeruginosa* in the lungs (109). The mitochondrial acid sphingomyelinase (ASM) is the key mediator in this process. ASM hydrolyzes sphingomyelin into ceramide, the latter plays a role in activating the apoptotic cascade (111, 112). Ceramide, together with the mitochondrial cytochrome c, triggering apoptosis in neutrophils. Neutrophils lacking ASM are resistant to pyocyanin-induced cell death, suggesting that ASM is crucial for mediating this process (107). Neutrophil apoptosis severely impairs the ability of the body to clear bacterial infection and likely contributes to the *P. aeruginosa* evasion of host immune defenses.

#### 2.2.2 Induction of NETosis

Neutrophils employ defense mechanism against pathogens through the formation of neutrophil extracellular traps (NETs). These NETs are intricate networks of chromatin fibers decorated with antimicrobial proteins (e.g., neutrophil elastase, cathepsin G, and histones that have a high affinity for DNA) released by neutrophils during a programmed cell death called NETosis (113, 114). NETs play a crucial role in the immune response by entrapping and killing a wide range of pathogens, including bacteria, fungi, viruses, and parasites (115, 116). NETs formation involves the decondensation of nuclear chromatin through histone citrullination by the peptidyl arginine deiminase IV (PAD4) (117–119). This process leads to the release of chromatin through a ruptured nuclear envelope, where it combines with cytosolic antimicrobial proteins and extracellular DNA (116, 120). Together, these components form the extracellular structures, which trap and neutralize pathogens. NETs released during NETosis are particularly important in trapping and killing bacterial pathogens such as *P. aeruginosa* and *Staphylococcus aureus* in pneumonia and chronic lung diseases like CF and COPD (121, 122). Pyocyanin has been identified as the first secreted bacterial toxin that enhances NET formation by stimulating an increase in the production of ROS within neutrophils (123). While NET plays a crucial role in combating microbial invaders, excessive NET formation can have adverse negative effects, where prolonged NET presence contributes to tissue damage and persistent inflammation. Due to the release of DNA and various enzymes that damage host tissues, NETs are highly pro-inflammatory. Persistent NETosis leads to the release of large amounts of ROS and pro-inflammatory molecules, creating a feedback loop of chronic inflammation (124). The overproduction of NETs in response to *P. aeruginosa* colonization can exacerbate neutrophilic inflammation (125), increasing airway obstruction and causing mucociliary dysfunction (126). In diseases such as CF and advanced stages of COPD where *P. aeruginosa* is a prominent pathogen, pyocyanin-induced NETosis is predicted to exacerbate the disease progression. The excessive release of NETs contributes to the formation of thick, viscous mucus, a hallmark of the disease (124, 126). Over time, the ongoing NETosis and chronic inflammation lead to irreversible lung damage and a decline in pulmonary function.

#### 2.2.3 Induction of macrophage dysfunction

Macrophages are crucial immune myeloid cells involved in both innate and adaptive immune responses (127–129). They originate from monocytes and are found throughout the body, acting as one of the first responders to infections (130). Macrophages phagocytize microbial pathogens, enclosing them in a phagosome that fuses with lysosomes, where microbes are destroyed by digestive enzymes and ROS (131, 132). *In vitro*, studies have shown that pyocyanin selectively disrupts the phagocytic function rather than overall macrophage impairment and dysfunction (133). Specifically, pyocyanin incapacitates macrophages to phagocytize apoptotic cells while maintaining their capacity to ingest inert particles such as latex beads. This impairment results in late apoptotic and necrotic cell accumulation in mouse tissues infected with pyocyanin-producing *P. aeruginosa*. In contrast, tissue infected with pyocyanin-deficient strains does not exhibit this accumulation (108). The failure of macrophages to clear apoptotic cells contributes to prolonged inflammation, as these unengulfed cells can undergo secondary necrosis, releasing inflammatory mediators that exacerbate tissue damage and promote a new cycle of inflammation (134, 135). The underlying mechanism of this impaired engulfment is linked to the disruption of the Rho GTPase signaling, which is crucial for phagocytosis by pyocyanin-generated ROS (108, 136). This disruption in the phagocytic clearance of apoptotic cells will likely cause chronic inflammation and weakened immune responses, potentially worsening tissue damage during infections.

#### 2.2.4 Induction of natural killer cells apoptosis

Natural killer (NK) cells are lymphocytes in the innate immune system that play a critical role in defending the body against infections and malignancies. Unlike T and B cells, NK cells do not require prior exposure to a pathogen to recognize and kill infected or abnormal cells (137, 138). They possess the ability to detect stressed, infected, or cancerous cells through a balance of activating and inhibitory receptors (139). NK cells respond rapidly to infections by releasing cytotoxic molecules like perforin and granzymes, which induce apoptosis and necrotic cell death in target cells. In addition, NK cells produce cytokines such as interferon-gamma (IFN-γ), which helps activate other immune cells (140). While NK cells are traditionally associated with viral infections and tumor surveillance, they also play an important role in bacterial infections (137, 141). NK cells can directly kill bacteria-infected cells and secrete cytokines that enhance the response of macrophages and other immune cells. A key example of this is the production of IFN-γ by NK cells, which activates macrophages to enhance bacterial killing via phagocytosis and ROS production (142, 143). Pyocyanin was shown to induce apoptosis in the human NK cell line, NK92. Interestingly, studies have shown that pyocyanin-producing *P. aeruginosa* are more effective at inducing apoptosis in NK92 cells compared to the pyocyanin-deficient strain (144). This apoptosis is primarily mediated through mitochondrial damage, although interesting, ROS generated by pyocyanin do not appear to play a significant role in this process (145). The excessive NK cell apoptosis induced by pyocyanin may have serious implications for immune function, potentially leading to reduced immune surveillance and responsiveness. Consequently, this could increase the susceptibility to infections and highlight the importance of pyocyanin as a virulence factor in *P. aeruginosa* pathogenicity.

#### 2.2.5 Pyocyanin-regulated cytokine expression

Pyocyanin-mediated oxidative stress has been shown to trigger the expression of pro-inflammatory cytokines through the activation of various host signaling pathways, among others, NF-κB and MAPK (93, 146–148). Under normal conditions, the production of cytokines such as IL-6, TNF-α, IL-1β, and IL-8 is crucial in the recruitment of immune cells, such as neutrophils and macrophages, to the site of infection and eliminating pathogens (98, 149–153). However, the overproduction or dysregulation of these cytokines induced by pyocyanin can lead to persistent inflammation, contributing to chronic inflammatory diseases such as CF and advanced stages of COPD. To better understand the consequences of pyocyanin-mediated inflammation, we highlighted the differential effects of this toxin on acute versus chronic inflammation. In acute inflammation, cytokine production is typically a short-term response aimed at combating infection and facilitating healing (154). However, when there is an excessive or sustained cytokine response, the inflammatory process becomes dysregulated and transition into chronic inflammation (155). This transition is a hallmark of chronic lung diseases such as CF and advanced stages of COPD, where prolonged *P. aeruginosa* colonization leads to a cycle of continuous immune activation and tissue damage.

Microarray analysis of airway epithelial cells exposed to pyocyanin has revealed several genes involved in inflammatory processes that are upregulated, including IL-6, TNF-α, IL-1β, IL-8, granulocyte colony-stimulating factor (G-CSF), granulocyte-macrophage colony-stimulating factor (GM-CSF) and CXCL1 (93). These cytokines and growth factors have significant roles in both innate and adaptive immunity. G-CSF and GM-CSF are critical for the activation and proliferation of granulocytes and macrophages, which are essential for the initial defense against infection. CXCL1 is a potent neutrophilic chemokine. As mentioned above, however, when produced in excess, these proinflammatory mediators exacerbate tissue damage, further perpetuating the proinflammatory cycles (156–158).

Acute inflammation is typically short-lived and resolved once the pathogen is eliminated. However, in the presence of pyocyanin, this initial acute response can be disrupted and transitioned to chronic inflammation. Pyocyanin induces the secretion of IL-23 to promote the differentiation and activation of Th17 cells, a subset of T-helper cells that play a key role in sustaining chronic inflammatory states (93, 159). During chronic inflammation, the immune system fails to eliminate infecting pathogens and leading to persistent inflammation. This phenomenon is particularly pronounced in diseases like CF and advanced stages of COPD, where chronic airway infection by *P. aeruginosa*, results in a vicious cycle of immune activation, tissue damage, and disease progression (160, 161). The impact of pyocyanin on both acute and chronic inflammation emphasizes the need for targeted therapeutic strategies that can modulate the inflammatory response and mitigate the harmful effects of pyocyanin on host tissues.

### 2.3 Disruption of airway epithelial mucosal immunity functions

The cystic fibrosis transmembrane conductance regulator (CFTR) is an anion channel that allows ion flux, such as chloride (Cl^-^), at the apical side of airway epithelia (162–164). Increased chloride secretion is immediately followed by water movement. This process helps maintain the volume of airway surface liquid (ASL) and ensures the proper hydration of mucus. Proper Cl^-^ transport ensures that the mucus layer covering the airways remains adequately hydrated, allowing optimal mucociliary clearance of bacteria and foreign particles (165–167). However, defective CFTR leads to dehydrated and thickened mucus, a hallmark of CF (162). Mucociliary clearance is a critical component of the innate defense system in the airway, protecting the respiratory tract by trapping inhaled pathogens, allergens, and particulate matter, which are removed through the mucociliary machinery (79, 168–170). However, mucus secretion is often dysfunctional, and mucociliary transport machinery is disabled in many chronic respiratory diseases (170–172). Goblet cell hyperplasia, metaplasia, and excessive mucus production are key pathological features of chronic airway diseases such as chronic obstructive bronchitis (part of COPD) and CF. Chronic respiratory infections often worsen these conditions, particularly with *P. aeruginosa*, which leads to persistent inflammation and increased mucus hypersecretion (173). Studies suggest that Cl^-^ transport by CFTR is affected by pyocyanin-generated ROS production, which leads to glutathione and ATP depletion. Consequently, CFTR function is inhibited, impairing chloride transport in airway epithelia (174). Furthermore, pyocyanin has been found to disrupt CFTR Cl^-^ transport by inhibiting the endocytic recycling of CFTR and inactivating the airway epithelial vacuolar ATPase, which reduces the expression and trafficking of CFTR, hence disrupting Cl^-^ transport (175, 176). Excessive mucus clogs the airways, creating a favorable niche for microbial colonization and infection. Disruption of ciliary function by pyocyanin (see Section 2.1.1) further deteriorates mucociliary clearance of thickened mucus, leading to microbial-mediated acute exacerbation often seen in CF and COPD (177, 178).

**Figure 3:**
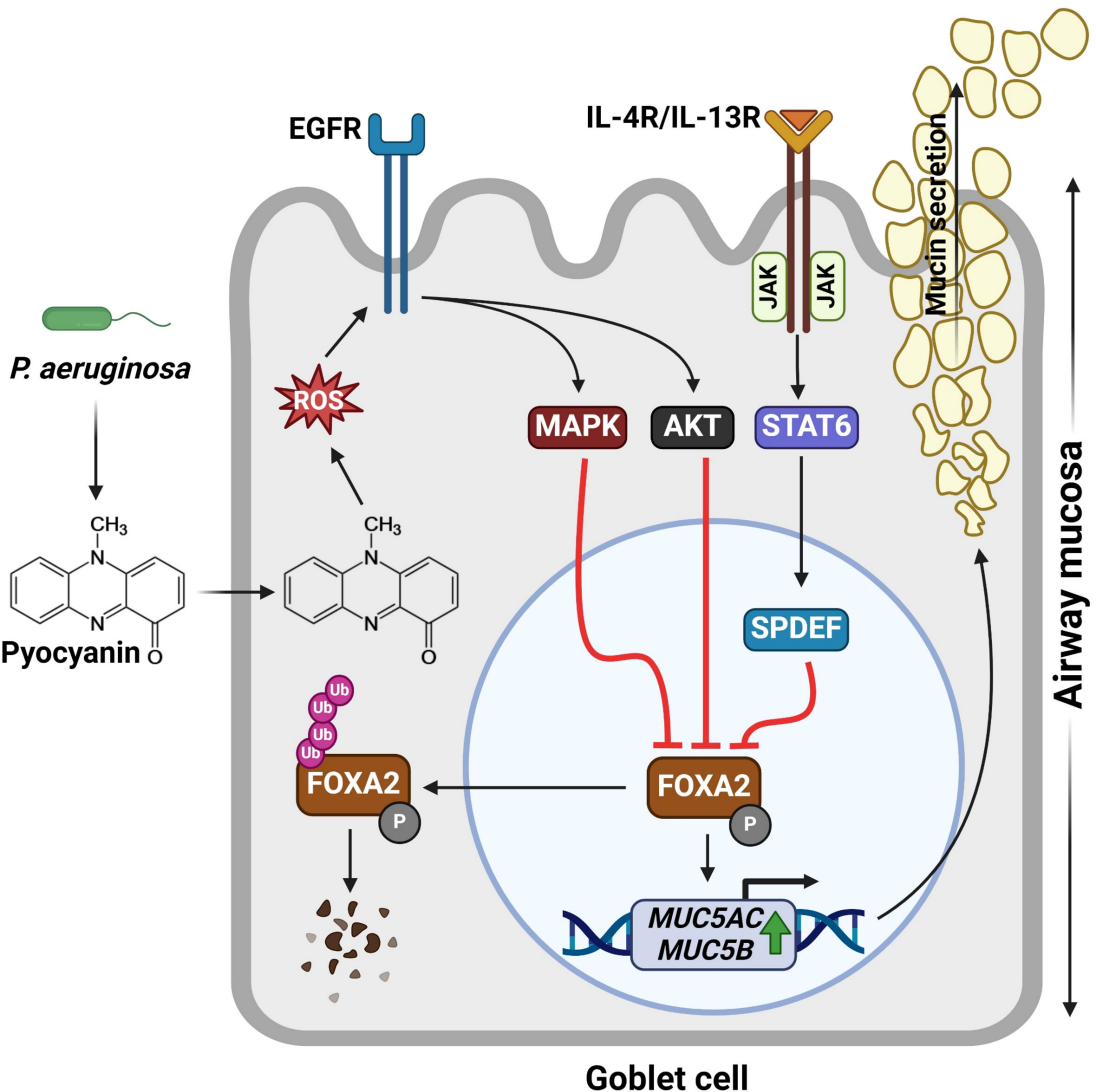
Induction of the signaling pathways by pyocyanin that drives mucus hypersecretion in diseased airways. IL-4R/IL-13R and EGFR activation by pyocyanin trigger JAK-STAT6-SPDEF, MAPK, and AKT signaling pathways, respectively. These pathways convergently suppress the expression of FOXA2, a transcription factor that regulates airway mucus homeostasis. Posttranslational phosphorylation sequesters phosphorylated FOXA2 to the cytoplasm destined for ubiquitination and degradation. The loss of FOXA2 results in the increased expression of the *MUC5AC* and *MUC5B* mucin genes, leading to goblet cell metaplasia, hyperplasia, and mucin overproduction. Figure generated with BioRender.com.

Multiple studies conducted by our laboratory have demonstrated that chronic exposure to pyocyanin contributes to goblet cell metaplasia and hyperplasia, mucus hypersecretion (26, 173, 179–182), as well as emphysema (41). Pyocyanin inhibits the expression of Forkhead box protein A2 (FOXA2), a key transcription factor that regulates lung alveolarization and airway mucus homeostasis (183). In healthy airways, FOXA2 negatively regulates goblet cell development, maintaining a balance between the need for mucus production and the prevention of excessive secretion (183, 184). Pyocyanin inactivates FOXA2 via the activation of EGFR-AKT/ERK1/2 pathways and the IL-4/IL-13-STAT6-SAM-pointed domain–containing Ets-like factor (SPDEF) pathways (26, 179, 180, 185, 186) (**Figure 3**). Activated EGFR-AKT/ERK1/2 and IL-4/IL-13-STAT6-SPDEF convergently suppress FOXA2, in which the stability and transcriptional activity of FOXA2 are regulated through various posttranslational modifications. Phosphorylation of FOXA2 by AKT sequesters the modified protein from nuclei to cytoplasm, where it is ubiquitinated and degraded (26, 187–189), increasing the expression of airway mucin genes *MUC5AC* and *MUC5B* (180, 190, 191). The balance between the airway gel-forming mucin MUC5AC and MUC5B is a key factor in both COPD and CF, influencing disease progression and severity​. In COPD patients, increased MUC5AC concentrations are more reliably associated with disease manifestations compared to MUC5B. Conversely, in CF patients, both MUC5AC and MUC5B are elevated, with a 30-fold increase for MUC5AC and an 8-fold increase for MUC5B, leading to a significantly higher MUC5AC/MUC5B ratio compared to healthy individuals. These distinct mucin profiles highlight the potential of MUC5AC and MUC5B, and especially their ratio, as biomarkers and therapeutic targets for managing these chronic respiratory diseases (192, 193). Additionally, ROS/RNS generated by pyocyanin modifies FOXA2 post-translationally, further contributing to mucus overproduction (26). These combined dysregulations lead to unchecked goblet cell proliferation, excessive mucus secretion, and pathological mucus accumulation observed in chronically diseased lungs infected by pyocyanin-producing *P. aeruginosa*.

The impact of pyocyanin on mucus hypersecretion is further intensified by its influence on the immune system, particularly by inducing Th2 cytokines secretion, such as IL-4 and IL-13 (41, 93). The IL-4/IL-13-STAT6-SPDEF pathway drives the Th2 response and goblet cell differentiation (93, 194). In a mouse chronic exposure model, pyocyanin polarizes an initially Th1 immune response towards the IL-4 and IL-13-dominated Th2 response, activating the IL-4/IL-13 receptor-STAT6-SPDEF signaling pathway (186, 195). SPDEF is responsible for triggering goblet cell metaplasia where airway epithelial multipotent basal, club, and ciliated cells are differentiated into mucus-secreting goblet cells and trigger a surge in mucus production (196, 197). Also, chronic oxidative stress causes a buildup of misfolded proteins in the endoplasmic reticulum (ER), triggering ER stress and activating the unfolded protein response (UPR) that intensifies mucus production and contributes to the development of chronic inflammation (198–200).

In CF and COPD where *P. aeruginosa* is a common and persistent colonizer, the consequences of pyocyanin-induced mucus hypersecretion are particularly severe (173). The resulting thick, sticky mucus not only obstructs airways but also creates an optimal environment for bacterial proliferation (201, 202), which in turn perpetuates inflammation and further increases mucus production. Consequently, this cascade promotes persistent infections and worsening lung function over time (203), which are hallmarks of diseases like CF and COPD.

### 2.4 Potential therapeutic approaches targeting pyocyanin as an anti-*P. aeruginosa* therapy

Antibiotics are most commonly prescribed for bacterial infections. Poor antimicrobial stewardship has led to the rise of antibiotic resistance (204, 205). Various preclinical strategies have been experimented to disrupt the non-essential aspect of bacterial life cycle, e.g., targeting non-growth bacterial virulence, reducing the risk of resistance development and preserving the beneficial microbiota, making it a sustainable option for long-term infection management (**Table 1**). One approach involves antioxidant therapies aimed at neutralizing the ROS generated by pyocyanin or boosting endogenous antioxidant defenses such as glutathione (206). N-acetylcysteine (NAC), a well-known antioxidant, has been studied for its ability to protect cells from pyocyanin-induced oxidative damage (207–209). Another strategy focuses on inhibiting pyocyanin production itself. Raloxifene, an oestrogen receptor modulator, has been shown to target PhzB2 in the phenazine biosynthetic pathway, is capable to reduce the virulence of *P. aeruginosa* (210). As discussed in Section 2.3, pyocyanin disrupts FOXA2 expression, a regulator of mucus homeostasis. The incretin mimetic Exendin-4 has been shown to restore FOXA2 expression by activating the GLP1R-PKA-PPAR-γ-dependent phosphatases PTEN and PTP1B, which dephosphorylate key kinases in pro-goblet cell EGFR-AKT/ERK1/2 and IL-4/IL-13-STAT6-SPDEF signaling pathways, lowering mucin production, and reduce *P. aeruginosa* burden in mouse models, offering a promising therapeutic strategy for managing mucus in these conditions (173). Other studies have revealed that N-Aryl Malonamides (NAMs) are potent inhibitors of the QS transcriptional regulator, MvfR (PqsR). In a murine model, NAMs were shown to protect intestinal barrier function, prevent bacterial dissemination, and reduce inflammatory cytokines. Inhibition of MvfR by NAMs reduces the production of multiple secondary metabolites, including pyocyanin, demonstrating the potential efficacy of this class of drugs in reducing *P. aeruginosa* pathogenicity (211). Meta-bromo-thiolactone (mBTL), an analog of the *P. aeruginosa* QS auto-inducers 3OC12–HSL (which activates LasR) and C4–HSL (which activates RhlR), inhibits pyocyanin production, biofilm formation, and protects the immortalized human alveolar-type II-like A549 cells and the invertebrate host *C. elegans* from *P. aeruginosa* infection (212, 213). Paraoxonase (PON) is a mammalian enzyme that breaks down lactones. It has been demonstrated that the expression of PON2 with intact lactonase activity facilitates 3OC12-HSL degradation in human airway epithelial cells and murine tracheal epithelial cells, suggesting that PON can disrupt the *P. aeruginosa* QS system by degrading the QS signaling molecule 3OC12-HSL (214, 215). Separately, research has focused on identifying the inactivation of a *P. aeruginosa* quorum-sensing signal by human airway epithelia. Quorum quenching (QQ) enzymes may potentially prevent pyocyanin synthesis by disrupting the bacterial QS system that regulates its production. These enzymes, such as AHL lactonases and acylases, degrade the signaling molecules responsible for QS, inhibiting the coordinated expression of pyocyanin and other virulence factors. By reducing pyocyanin levels, QQ enzymes may mitigate the oxidative damage and tissue toxicity caused by pyocyanin (216, 217). Additionally, immunomodulation strategies are being explored to restore the ability of the immune system to fight infections. These include protecting neutrophil, macrophage, and T-cell functions from pyocyanin-mediated toxicity could boost *P. aeruginosa* clearance (218, 219). By combining antioxidant treatments, pyocyanin biosynthesis inhibition, and immunomodulation, these strategies may restore immune function, mitigate oxidative stress, and ultimately reduce the lung damage associated with chronic infections.

**Table 1:** Classification of anti-pyocyanin drugs.

Drug	Classification	Mechanism	References
N-acetylcysteine	Antioxidant	ROS neutralization	(207–209, 216)
Glutathione	Antioxidant	Free radical scavenger	(206)
Raloxifene	Pyocyanin biosynthesis inhibitor	Disruption of pyocyanin biosynthesis pathway	(210)
Meta-bromo-thiolactone	Pyocyanin biosynthesis inhibitor	*P. aeruginosa* quorum sensing autoinducer analogs	(212, 213)
N-Aryl Malonamides	*P. aeruginosa* Quorum sensing inhibitor	Inhibition of the QS transcriptional regulator, MvfR	(211)
AHL lactonases and acylases	Quorum quenching enzymes	Degradation of the QS signaling molecules	(216, 217)
Paraoxonase	Quorum quenching enzymes	Degradation of 3OC12-HSL	(214, 215)
Exendin-4	Incretin mimetics	Inactivation of pyocyanin mediated pro-goblet cell signaling pathway	(173)

## 3 Conclusion

**Figure 4:**
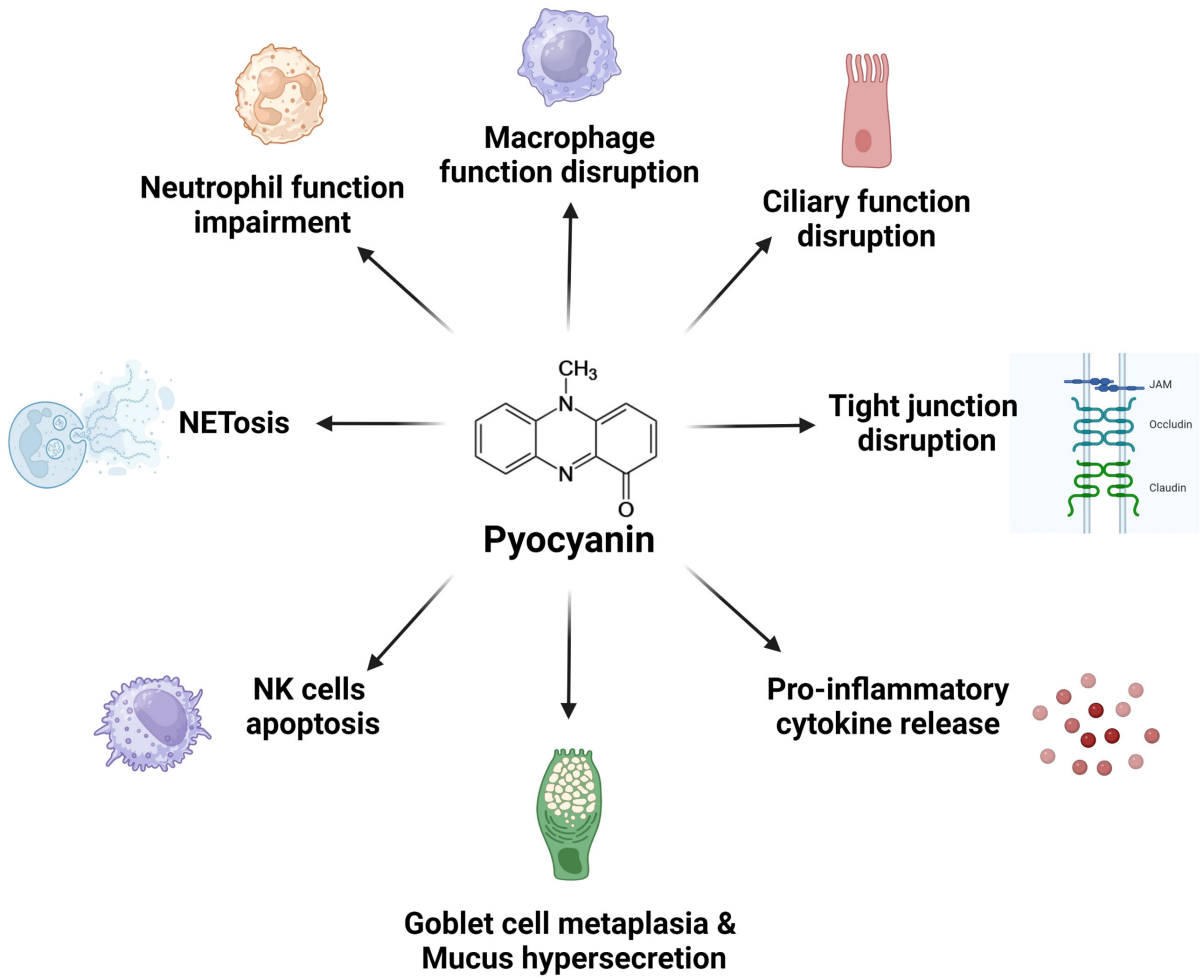
The multifaceted impact of pyocyanin on lung cell function. Pyocyanin disrupts multiple cellular functions in the lung, leading to inflammation, tissue damage, and impaired immune response. Collectively, these effects contribute to the severity of *P. aeruginosa* infections, particularly in individuals who are immunocompromised or with underlying lung conditions. Figure generated with BioRender.com.

Pyocyanin plays a crucial role in chronic lung infections by *P. aeruginosa* (25, 220). Pyocyanin contributes to persistent infections and chronic inflammation by inducing oxidative stress and modulating the host airway epithelial physiology and immune response. Its ability to generate ROS leads to cellular damage, further exacerbating inflammatory responses and impairing the natural defense mechanisms of the lung (**Figure 4**). Understanding the molecular mechanisms by which pyocyanin manipulates host cell functions are vital for developing targeted therapeutic approaches to mitigate its harmful effects and improve patient outcomes.
