# Corrigendum: Modulation of pulmonary immune functions by the *Pseudomonas aeruginosa* secondary metabolite pyocyanin

**DOI:** 10.3389/fimmu.2025.1601361

**Published:** 2025-04-09

**Authors:** Shi Qian Lew, Sook Yin Chong, Gee W. Lau

**Affiliations:** Department of Pathobiology, University of Illinois at Urbana-Champaign, Urbana, IL, United States

**Keywords:** *Pseudomonas aeruginosa*, pyocyanin, reactive oxygen and nitrogen species, oxidative stress, immune modulation, chronic lung diseases

In the published article, the reference for 39 was incorrectly written as “Thees A, Tan M-W, Le L, Wong SM, Tompkins RG, Calderwood SB, et al. PmtA regulates pyocyanin expression and biofilm formation in Pseudomonas aeruginosa. *Front Microbiol.* (2021) 12:789765. doi: 10.3389/fmicb.2021.789765”. It should be “Thees A, Pietrosimone K, Melchiorre C, Marden J, Graf J, Lynes M, et al. PmtA regulates pyocyanin expression and biofilm formation in Pseudomonas aeruginosa. *Front Microbiol.* (2021) 12:789765. doi: 10.3389/fmicb.2021.789765”.

The authors apologize for this error and state that this does not change the scientific conclusions of the article in any way. The original article has been updated.

